# Prenatal Restraint Stress Generates Two Distinct Behavioral and Neurochemical Profiles in Male and Female Rats

**DOI:** 10.1371/journal.pone.0002170

**Published:** 2008-05-14

**Authors:** Anna Rita Zuena, Jerome Mairesse, Paola Casolini, Carlo Cinque, Giovanni Sebastiano Alemà, Sara Morley-Fletcher, Valentina Chiodi, Luigi Giusto Spagnoli, Roberto Gradini, Assia Catalani, Ferdinando Nicoletti, Stefania Maccari

**Affiliations:** 1 Perinatal Stress Lab., University Lille 1, Villeneuve d'Ascq, France; 2 Department of Human Physiology and Pharmacology, University of Rome “La Sapienza”, Rome, Italy; 3 Istituto Neurologico Mediterraneo Neuromed, Pozzilli, Italy; 4 Institute of Anatomic Pathology, Tor Vergata University, Rome, Italy; 5 Department of Experimental Medicine, University of Rome “La Sapienza”, Rome, Italy; National Institutes of Health, United States of America

## Abstract

Prenatal Restraint Stress (PRS) in rats is a validated model of early stress resulting in permanent behavioral and neurobiological outcomes. Although sexual dimorphism in the effects of PRS has been hypothesized for more than 30 years, few studies in this long period have directly addressed the issue. Our group has uncovered a pronounced gender difference in the effects of PRS (stress delivered to the mothers 3 times per day during the last 10 days of pregnancy) on anxiety, spatial learning, and a series of neurobiological parameters classically associated with hippocampus-dependent behaviors. Adult male rats subjected to PRS (“PRS rats”) showed increased anxiety-like behavior in the elevated plus maze (EPM), a reduction in the survival of newborn cells in the dentate gyrus, a reduction in the activity of mGlu1/5 metabotropic glutamate receptors in the ventral hippocampus, and an increase in the levels of brain-derived neurotrophic factor (BDNF) and pro-BDNF in the hippocampus. In contrast, female PRS rats displayed reduced anxiety in the EPM, improved learning in the Morris water maze, an increase in the activity of mGlu1/5 receptors in the ventral and dorsal hippocampus, and no changes in hippocampal neurogenesis or BDNF levels. The direction of the changes in neurogenesis, BDNF levels and mGlu receptor function in PRS animals was not consistent with the behavioral changes, suggesting that PRS perturbs the interdependency of these particular parameters and their relation to hippocampus-dependent behavior. Our data suggest that the epigenetic changes in hippocampal neuroplasticity induced by early environmental challenges are critically sex-dependent and that the behavioral outcome may diverge in males and females.

## Introduction

Prenatal Restraint Stress (PRS) in rats is a validated model of early stress with permanent behavioral and neurobiological consequences [1], [2]. Although the existence of sexual dimorphism in the outcome of PRS was hypothesized more than 30 years ago [3], only a few studies have addressed this issue since then. Recent evidence indicates that male rats subjected to PRS (“PRS rats”) are more prone to developing learning impairments than female PRS rats [4]. It is worthy noting, however, that data on PRS and cognitive impairment are not unequivocal with some authors reporting a reduction in the performance of juvenile and adult PRS male rats in the water maze [5], [6], and others reporting no effect of PRS on spatial and working memory [7]. In addition, Vallée et al. [8] have shown that PRS rats display learning impairments during aging but not in adulthood. Data on the effect of PRS on anxiety-like behavior are also conflicting. There are two reports of increased anxiety-like behavior in adult female, but not male, PRS rats [6], [7]. In contrast, an increased response to anxiogenic stimuli has been reported in adult male PRS rats using the elevated plus maze (EPM) [1], [9] and the open field test [10]. There is evidence that the hypothalamic-pituitary adrenal (HPA) axis response to stress is greater in female than in male PRS rats [4], [11], although PRS can switch the female response to stress into a male pattern, reducing the increase in corticosterone secretion induced by stress [7]. A more integrated study in which behavior is examined along with its putative neurobiological correlates may better clarify the gender effect in the outcome of PRS. We focused on the hippocampus, which is an established target brain region for PRS [5], [12]–[15]. Within the hippocampus, we examined (i) the expression and activity of metabotropic glutamate (mGlu) receptors; (ii) levels of brain-derived neurotrophic factor (BDNF); and (iii) neurogenesis in the dentate gyrus. mGlu receptors form a family of eight subtypes subdivided into three groups on the basis of amino acid sequence, pharmacological profile, and transduction mechanisms. Group I includes mGlu1 and mGlu5 receptors, which are coupled to polyphosphoinositide (PI) hydrolysis; group-II (mGlu2, mGlu3); and group-III (mGlu4, mGlu6, mGlu7, and mGlu8) receptor subtypes are instead coupled to Gi proteins in heterologous expression systems [16], [17]. At least group-I and group-II mGlu receptors are widely implicated in learning and memory processes [18], [19] and are potential targets for the treatment of anxiety disorders [20]–[22]. Changes in hippocampal BDNF levels and neurogenesis occur during learning and in response to stress, and are intimately associated with the pathophysiology of mood and anxiety disorders [23]–[29]. mGlu receptors, BDNF levels and neurogenesis are tightly interconnected. mGlu receptors are expressed by neural stem cells, and regulate the proliferation, differentiation, and survival of these cells [30], [31]; BDNF is required for basal neurogenesis and mediates the enhancement of neurogenesis in response to exercise, dietary restrictions and psychotropic drugs [32]–[35]; and distinct mGlu receptor subtypes regulate BDNF expression and BDNF receptor signaling in brain tissue [36]–[40].

Here, we report that PRS has different, sometimes opposite, effects in male and female rats in terms of spatial learning, anxiety, expression and activity of group-I and group-II mGlu receptors in the hippocampus, hippocampal BDNF levels, and adult neurogenesis in the hippocampal dentate gyrus.

## Results

### Sex specific effects of PRS on anxiety-like behavior and spatial learning

**Figure 1:**
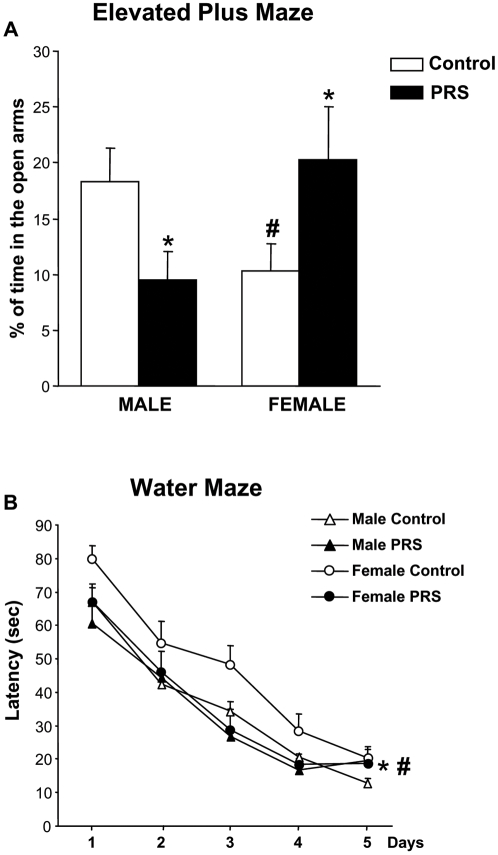
Changes in anxiety-like behavior and spatial learning induced by PRS in male and female rats. The time spent by control and PRS rats in the open arms of the EPM is shown in (A). No changes in total locomotor activity were detected among groups (data not shown). There was a clear-cut gender effect of PRS in the EPM, ANOVA group×sex interaction: F _(1,28)_ = 9.92, p<0.05. Values are expressed as means±S.E.M. (n = 8 rats per group). (#) p<0.05 control males vs control females; (*) p<0.05 PRS vs. control animals of each sex (ANOVA+Newman-Keul's *post-hoc* test). The latency in finding the hidden platform in the Morris water maze is shown in (B) All groups of animals (control and PRS rats of both genders) were able to learn the task through consecutive trials and days of testing (ANOVA for repeated measure: trials effect F _(4-924)_ = 24.06, p<0.05; days effect: F _(4-924)_ = 127.55, p<0.05). However, ANOVA also revealed that the performance of control female rats was poorer than that of control male rats (# p<0.05: ANOVA sex effect F _(1-924)_ = 4.46 and Newman-Keul's *post-hoc* test). PRS improved learning in female rats (* p<0.05: ANOVA group effect F _(1-924)_ = 4.46, control females vs all other groups, Newman-Keul's *post-hoc* test), but failed to affect learning in male rats.

Anxiety-like behavior was measured in the Elevated Plus Maze (EPM); the percentage of time spent in the open arms of the maze was considered a measure of anxiety since no difference in total locomotor activity was detected among groups (data not shown). The test revealed that female control rats (i.e. rats that had not been exposed to PRS) spent less time than male controls in the open arms, reflecting increased anxiety-like behavior among control females (Fig. 1A). There was a clear-cut gender effect of PRS in the EPM. PRS increased the time spent in the open arms (i.e. reduced anxiety) in female rats, but had the opposite effect (i.e. increased anxiety) in male rats (Fig. 1A).

Spatial learning was assessed using the Morris water maze for five consecutives days. All groups of animals were able to learn the task through consecutive trials and days of testing. However, female control rats performed poorly when compared to male rats [see also 4], [6]. PRS improved learning in female rats, bringing their performance to the same level as males. In contrast, PRS failed to affect learning in male rats (Fig. 1B).

### PRS reduces the number of newly-formed cells within the dentate gyrus in male but not in female rats

**Figure 2:**
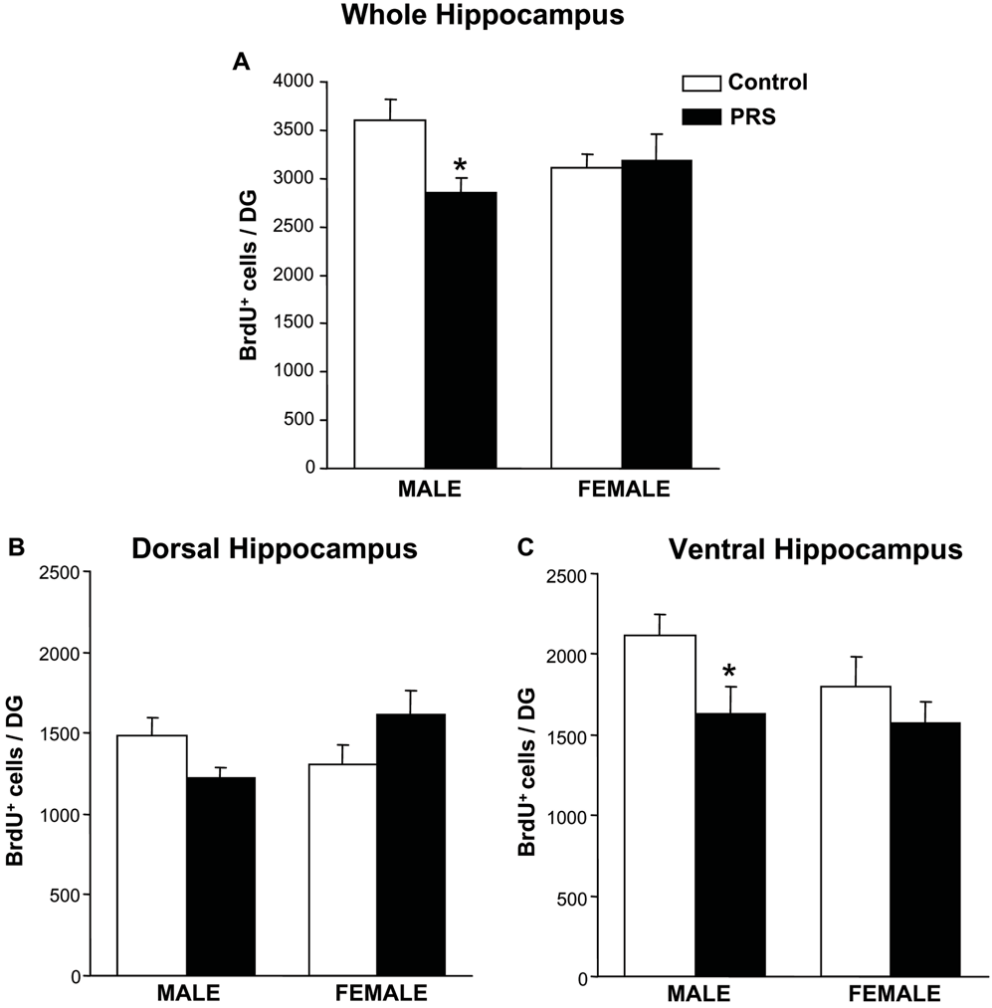
Gender effect of PRS on the survival of newly-formed cells in the hippocampal dentate gyrus. Data obtained in the whole hippocampus and in the dorsal and ventral hippocampus are shown in (A), (B), and (C), respectively. In the whole hippocampus the outcome of PRS was gender-dependent (ANOVA group×sex interaction: F _(1,16)_ = 4.53, p<0.05;). Values are means±S.E.M. of 5 measurements. * p<0.05 for PRS vs control males, ANOVA+Newman-Keul's *post-hoc* test

We measured the number of BrdU^+^ cells in the sub-granular zone (SGZ) of the dentate gyrus, a niche of persistent neurogenesis in the adult brain, and in the adjacent granular cell layer (GCL). Measurements were carried out 2 weeks after a 5-day treatment with BrdU, when BrdU immunostaining mainly reflects the survival of newly-formed cells. We found no difference in the number of BrdU^+^ cells between control male and female rats in the hippocampus as a whole, or in the ventral and dorsal portions of the hippocampus. However, the outcome of PRS on the steady-state survival of newly-formed cells was gender-dependent. In the whole hippocampus, PRS reduced the number of BrdU^+^ cells in male rats (Fig. 2A). This effect was accounted for by a significant reduction in the number of BrdU^+^ cells in the ventral hippocampus, whereas only a trend towards a reduction was seen in the dorsal hippocampus (Fig. 2B,C). In contrast, PRS did not affect the steady-state number of BrdU^+^ cells in the ventral and dorsal hippocampus of female rats, although a trend towards an increase was seen in the dorsal hippocampus (Fig. 2B,C).

### PRS does not affect differentiation of newly-formed cells into neurons or astrocytes in the hippocampal dentate gyrus

**Figure 3:**
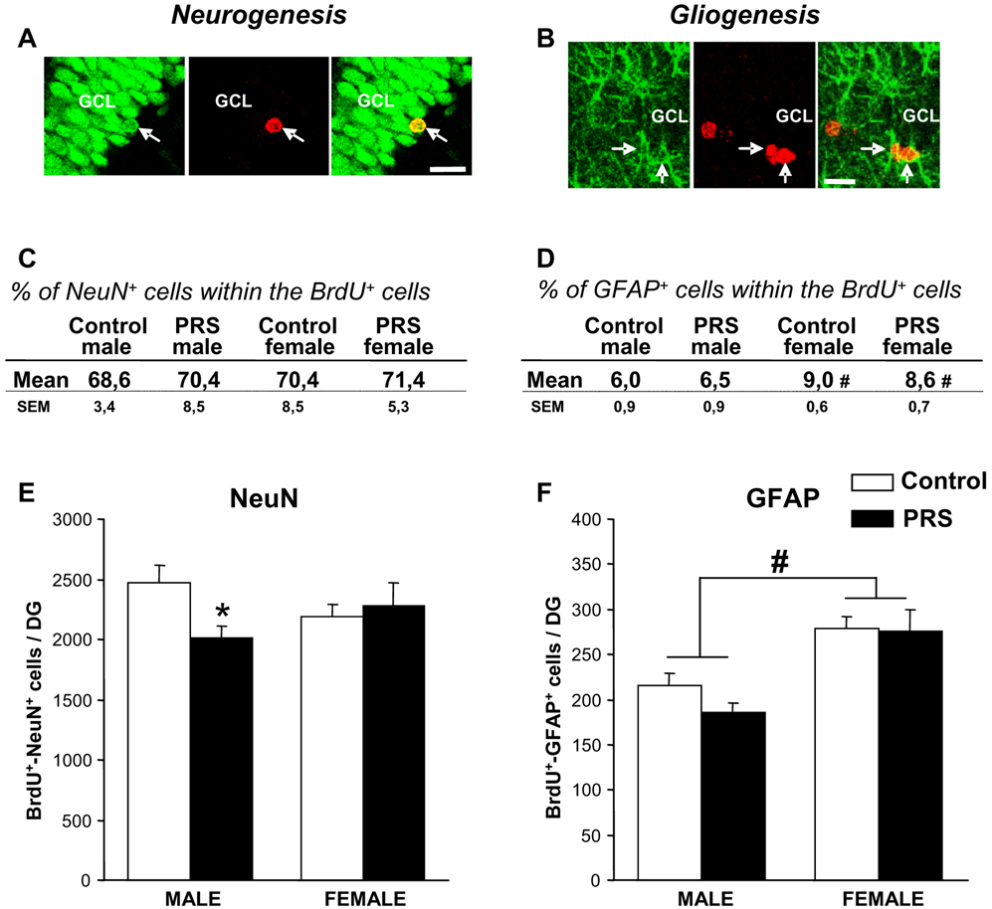
Hippocampal neurogenesis and gliogenesis in control and PRS rats. Representative confocal laser scanning microscope image showing cells double-labeled for BrdU (red nuclear staining) and the neuronal marker NeuN (green nuclear staining) (A), and cells double-labeled for BrdU and the glial marker GFAP (green cytoplasmic staining) (B). DG: dentate gyrus; GCL: granular cell layer; arrows: double labeled cells. Scale bars-20 µm. (C,D) PRS did not affect the percentage of NeuN^+^/BrdU^+^ cells or the percentage of GFAP^+^/BrdU^+^ cells within the total population of BrdU^+^ cells in male and female rats. It is worth noting, however, that both control and PRS female rats showed more efficient gliogenesis, as reflected by a greater percentage of GFAP^+^/BrdU^+^ cells than in male rats (# p<0.05 ANOVA sex effect: F _(1,16)_ = 6.48,). (E) PRS reduced the total number of BrdU^+^/NeuN^+^ cells in male rats (* p<0.05, ANOVA+Newman-Keul's *post-hoc* test), with no effect in female rats. (F) PRS had no effect on astroglial differentiation; female rats, independent of group, showed an increase in the total number of newly-formed astroglial cells (# p<0.05, ANOVA sex effect: F _(1,16)_ = 4,53). Bars represent means±SEM (n = 5 rats per group).

Newly-formed neurons and astrocytes were identified by double BrdU/NeuN or BrdU/GFAP immunolabeling (Fig. 3A,B). In male rats, PRS reduced the total number of BrdU^+^/NeuN^+^ cells (Fig. 3E), but did not change the percentage of NeuN^+^ cells within the total population of BrdU^+^ cells (Fig. 3C). Thus, the reduction in newly-formed neurons did not reflect an effect of PRS on cell differentiation, but was secondary to the overall reduction in BrdU^+^ cells. PRS did not change the number of NeuN^+^/BrdU^+^ cells in female rats (Fig. 3C,E), or the number of GFAP^+^/BrdU^+^ cells in either male or female rats (Fig. 3D,F). It is worth noting, however, that both control and PRS female rats showed more efficient gliogenesis, as reflected by a greater number and percentage of GFAP^+^/BrdU^+^ cells than in male rats (Fig. 3D,F).

### PRS increases hippocampal BDNF levels in male but not in female rats

**Figure 4:**
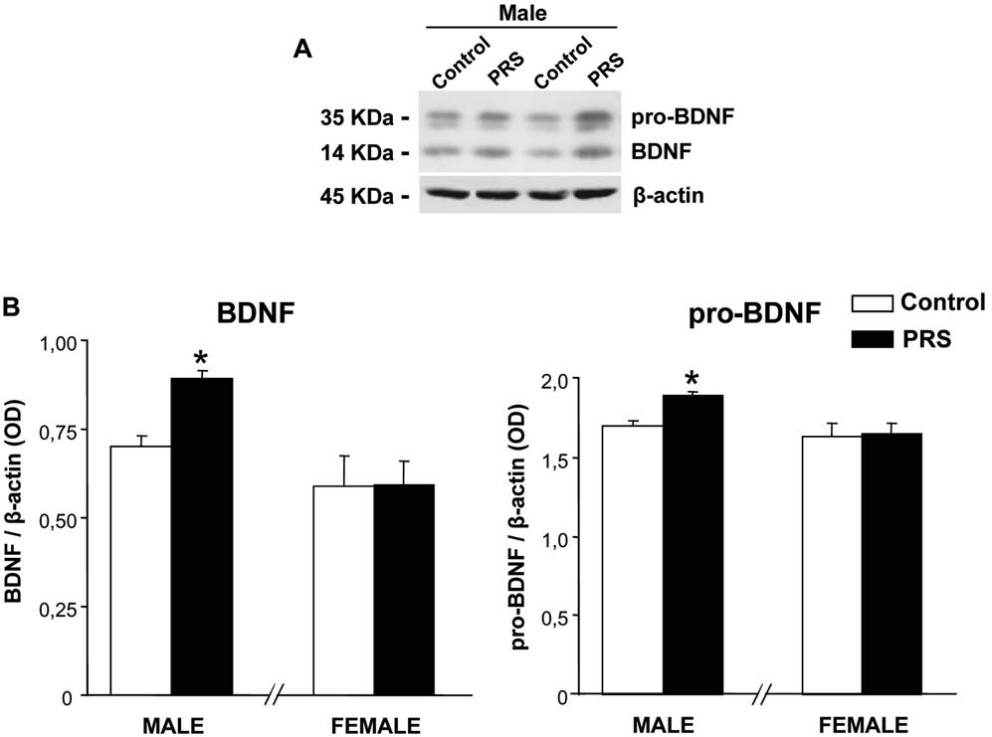
PRS enhances hippocampal BDNF levels in male rats. (A) Immunoblot analysis showed a 14 kDa band which corresponds to the mature form of BDNF, and a higher molecular weight band (about 35 kDa), which may correspond to its precursor, pro-BDNF. (B) PRS increased the steady-state levels of both BDNF and pro-BDNF in male rats (* p<0.05, ANOVA: F _(1,12)_ = 29.68, F _(1,12)_ = 27.09, respectively,), but had no effect in female rats. Male and female hippocampi were processed separately. Results are expressed as the ratio of the optical density (OD) of the pro-BDNF or BDNF band and the β-actin band. Values are expressed as means±S.E.M. (n = 7 rats per group).

We examined the expression of BDNF in the hippocampus by immunoblotting. The blot revealed a 14 kDa band which corresponds to the mature form of BDNF, and a higher molecular weight band (about 35 kDa), which may correspond to its precursor, pro-BDNF (Fig. 4A). PRS increased the steady-state levels of both BDNF and pro-BDNF in male rats, but had no effect in female rats (Fig. 4A,B). The increase in both BDNF and pro-BDNF suggests enhanced BDNF production/transcription/expression in the hippocampus of male rats exposed to PRS.

### Effects of PRS on the expression of group-I and -II metabotropic glutamate receptors in the hippocampus of male and female rats

**Figure 5:**
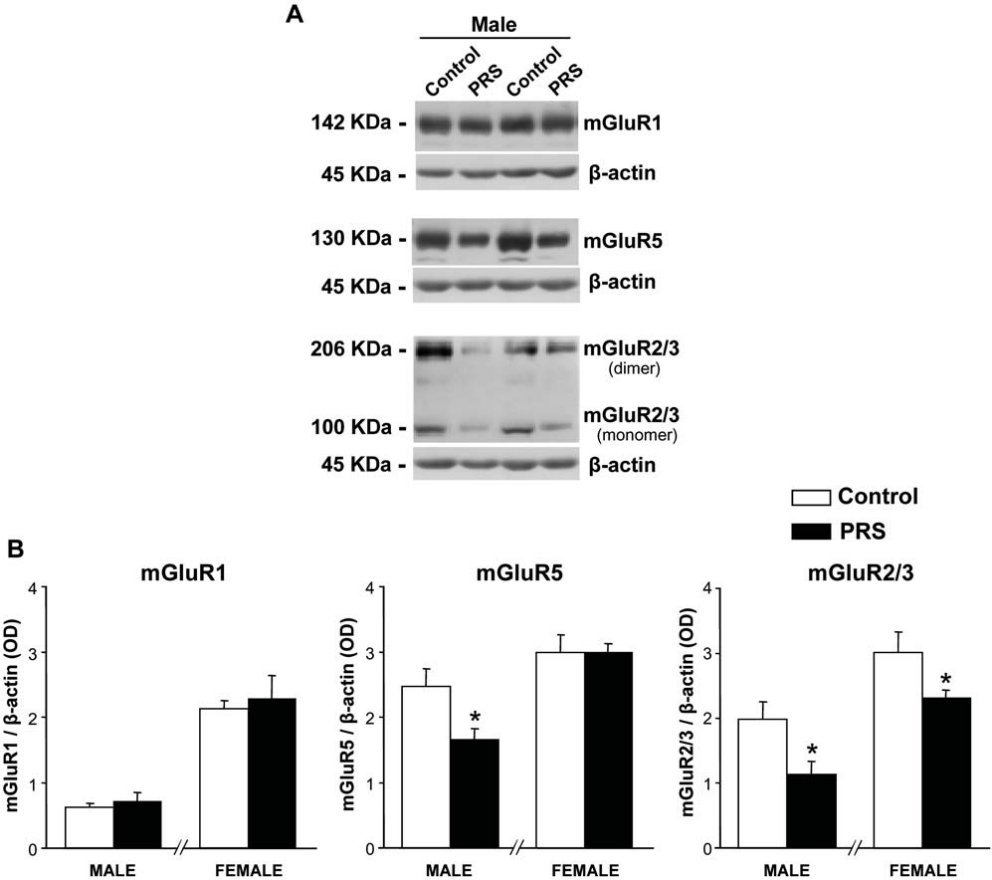
Expression of group-I and group–II mGlu receptors in the hippocampus of control and PRS rats. (A) Immunoblots with mGlu1a and mGlu5 receptor antibodies showed a 142 kDa and 130 kDa bands, respectively, corresponding to receptor monomers. No bands of higher molecular weight were detected under our experimental conditions. Blots with antibodies recognizing an epitope common to mGlu2 and mGlu3 receptors showed a 100 kDa band corresponding to receptor monomer(s), and a higher molecular weight band (206 kDa), which may correspond to receptor dimers. (B) In male rats, PRS induced a reduction in the expression of mGlu5 receptors (* p<0.05, ANOVA: F _(1,13)_ = 7.22,) but no change in the expression of mGlu1a receptors. PRS had no effect on the expression of mGlu1a and mGlu5 receptors in female rats. Interestingly, PRS reduced mGlu 2/3 receptor expression in the hippocampus of both male and female rats (ANOVA: F _(1,13)_ = 6.62, p<0.05 for males and F _(1,13)_ = 4.76, p<0.05 for females). Male and female hippocampi were processed separately. Results are expressed as the ratio of the optical density (OD) of the mGluR1a, mGluR5 or mGlu2/3 band and the β-actin band. Values are expressed as means±S.E.M. (n = 7 rats per group).

We examined the expression of mGlu1, mGlu5 and mGlu 2/3 receptor proteins in the whole hippocampus of male and female PRS rats, and their respective controls. Immunoblots with mGlu1a and mGlu5 receptor antibodies showed a 142 kDa and a 130 kDa band, respectively, corresponding to the receptor monomers (Fig. 5D). No bands of higher molecular weight were detected under our experimental conditions. Blots with antibodies recognizing an epitope common to mGlu2 and mGlu3 receptors showed a 100 kDa band corresponding to the receptor monomer(s), and a higher molecular weight band (206 kDa), which may correspond to receptor dimers (Fig. 5D). In male rats, PRS induced a reduction in the expression of mGlu5 receptors but did not affect the expression of mGlu1a receptors in the hippocampus (Fig. 5A,B). PRS had no effect on the expression of mGlu1a or mGlu5 receptors in female rats (Fig. 5A,B). Interestingly, PRS reduced mGlu 2/3 receptor expression in the hippocampus of both male and female rats (Fig. 5C).

### Sex-specific effects of PRS on mGlu receptor-mediated polyphosphoinositide (PI) hydrolysis in hippocampal slices

**Figure 6:**
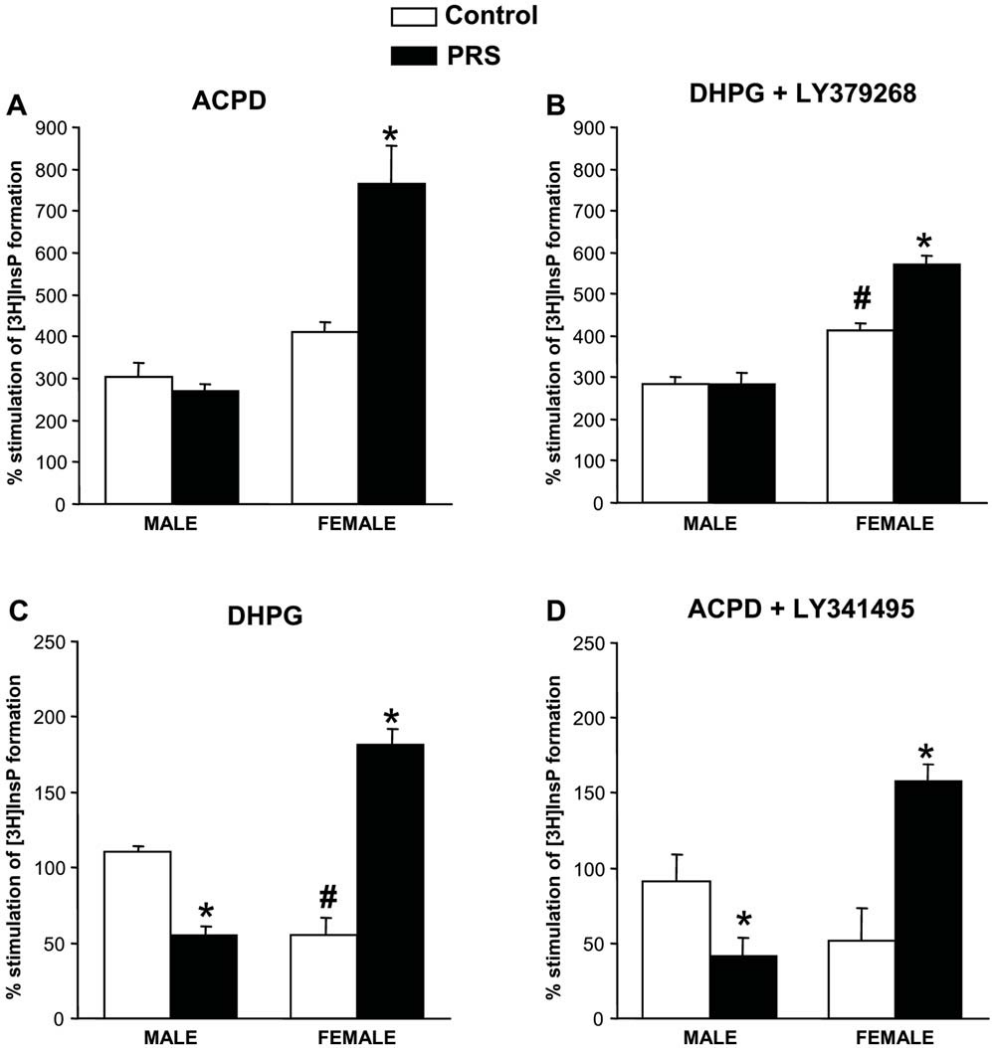
Gender effect in the effect of PRS on mGlu receptor-mediated PI hydrolysis in hippocampal slices. Stimulation of PI hydrolysis by the mixed mGlu receptor agonist, 1S,3R-ACPD (100 µM) (A), the selective mGlu1/5 receptor agonist, DHPG (200 µM) (C), DHPG combined with the selective mGlu2/3 receptor agonist, LY379268 (1 µM) (B), or 1S,3R-ACPD combined with the preferential mGlu2/3 receptor antagonist, LY341495 (1 µM) (D) in slices prepared from the whole hippocampus of male and female control and PRS rats. Values are expressed as means±S.E.M. of 9 individual measurements from 3 independent experiments. ANOVA for group×sex: F _(1,12)_ = 14.23, p<0.05 for ACPD; F _(1,12)_ = 14.30, p<0.05 for DHPG+LY379268; F _(1,12)_ = 97.12, p<0.05 for DHPG; F _(1,12)_ = 23.01, p<0.05 for ACPD+LY341495. * p<0.05 Newman-Keul's *post-hoc* test for PRS vs. control animals; # p<0.5 female vs. male rats.

We examined agonist-stimulated PI hydrolysis in slices prepared from the whole hippocampus. Using this model, the activation of group-I mGlu receptors directly stimulates PI hydrolysis, whereas activation of group-II mGlu receptors inactive *per se,* but amplifies the stimulation mediated by group-I mGlu receptors 41–43. Slices were challenged with 1S,3R-ACPD, a non subtype-selective mGlu receptor agonist that activates both group-I and group-II mGlu receptors, applied alone or in combination with LY341495, which preferentially antagonizes group-II mGlu receptors. Alternatively, slices were challenged with the selective group-I mGlu receptor agonist, DHPG, applied alone or in combination with the selective group-II mGlu receptor agonist, LY379268 [44 for a review]. 1S,3R-ACPD and DHPG were used at saturating concentrations of 100 µM and 200 µM, respectively; LY341495 and LY379268 were used at concentrations of 1 µM each. PRS differentially affected mGlu receptor-mediated PI hydrolysis in the two sexes. Stimulation of PI hydrolysis by ACPD or by DHPG *plus* LY379268, which reflects the combined activation of group-I and group-II mGlu receptors, was unchanged in PRS male rats, but was increased in PRS female rats (Fig. 6A,B). This increase in PRS female rats was entirely accounted for by the stimulation of group-I mGlu receptors because it was maintained in slices challenged with DHPG or with 1S,3R-ACPD combined with LY341495. Interestingly, these two treatments stimulated PI hydrolysis to a lower extent in control females than in control males, suggesting an even greater amplification by PRS in females (Fig. 6C,D). In contrast, stimulation of PI hydrolysis by DHPG or 1S,3R-ACPD combined with LY341495 (i.e. stimulation entirely mediated by group-I mGlu receptors) was substantially decreased in PRS male rats when compared to controls (Fig. 6C,D), an effect that was seen in response to 1S,3R-ACPD or DHPG with LY379268 (see above).

### Sex-specific effects of PRS on mGlu receptor-mediated PI hydrolysis in slices prepared from the dorsal and ventral hippocampus

**Figure 7:**
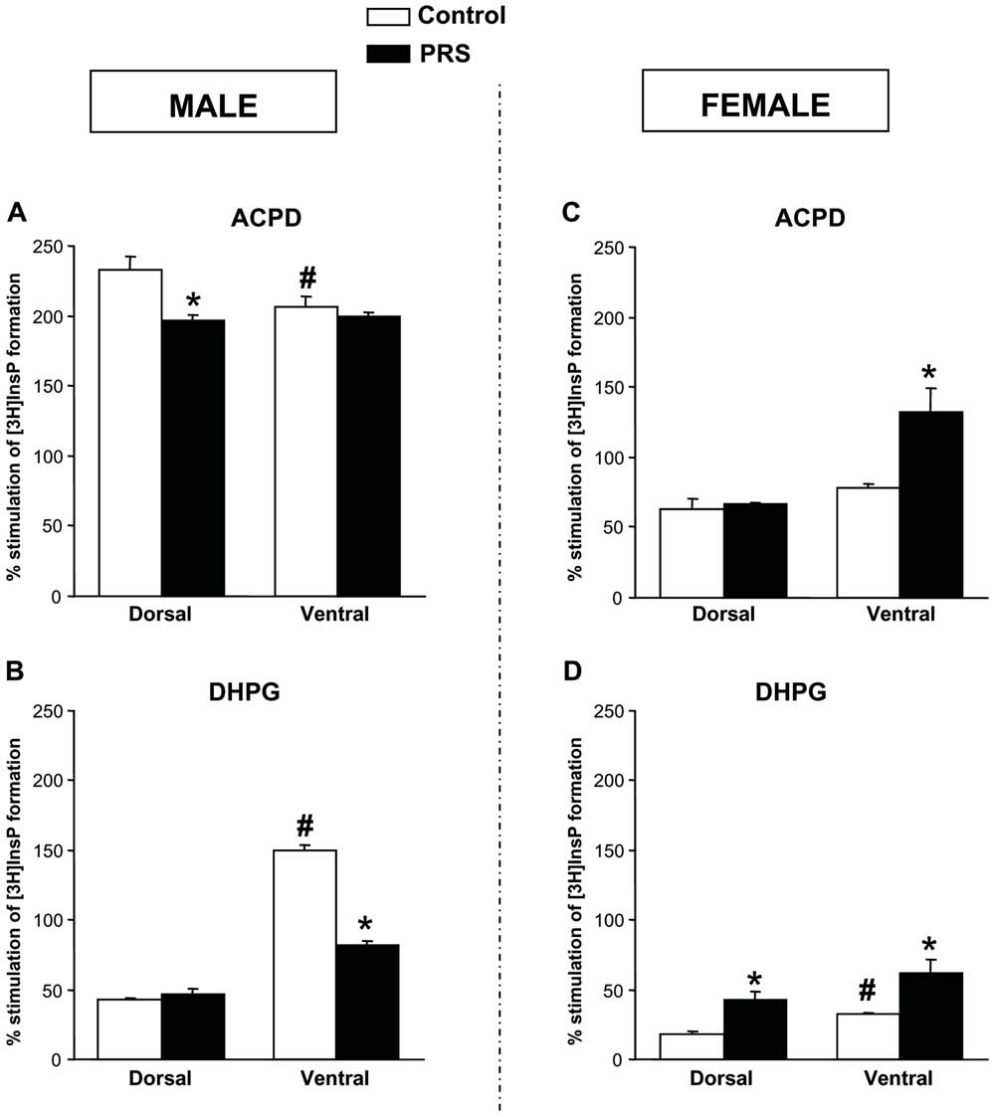
Gender effect in the effect of PRS on 1S,3R-ACPD or DHPG-stimulated PI hydrolysis in slices prepared from the dorsal or the ventral hippocampus. Data obtained from slices of male and female rat hippocampi are shown in (A,C) and (B,D), respectively. Values are expressed as means±S.E.M. of 6–9 measurements from 2–3 independent experiments. ANOVA for group×region: F_(1,8)_ = 4.97, p<0.05 for 1S,3R-ACPD and F_(1,8)_ = 128.79, p<0.05 for DHPG in males; F _(1,8)_ = 7.54, p<0.05 for 1S,3R-ACPD in females). * p<0.05 Newman-Keul's test *post-hoc* for PRS vs. control animals; # p<0.05 ventral vs. dorsal hippocampus (n = 5 rats per group).

We extended the examination above to slices prepared from the ventral or dorsal hippocampus because the ventral hippocampus is implicated in anxiety-like behavior, whereas the dorsal hippocampus is involved in spatial learning [reviewed in 45]. Slices were challenged with 1S,3R-ACPD alone or with DHPG. In the ventral hippocampus of male rats, PRS induced the same changes as were seen in the whole hippocampus, by reducing the PI hydrolysis stimulated by DHPG (i.e. hydrolysis entirely mediated by group-I mGlu receptors), without changing the extent of stimulation by 1S,3R-ACPD (an agonist of both group-I and group-II mGlu receptors) (Fig. 7A,B). Stimulation of PI hydrolysis in the male dorsal hippocampus was only slightly affected by PRS, which induced a small reduction in the efficacy of 1S,3R-ACPD, but no change in the efficacy of DHPG (Fig. 7A,B). In female rats, PRS selectively amplified the PI response to 1S,3R-ACPD in the ventral hippocampus, whereas it amplified the response to DHPG in both the ventral and dorsal hippocampus (Fig. 7C,D). It was not possible to compare responses between control males and control females because of technical difficulties involving multiple variables in the same experiment (ventral and dorsal hippocampus, control and PRS rats, males and females).

## Discussion

Our data demonstrate a clear gender effect on the outcome of PRS on hippocampal neuroplasticity and hippocampus-dependent behavior. Hippocampal neurons are highly plastic and respond to early environmental challenges with long-lasting changes in the mechanisms regulating synaptic plasticity and network organization, neurogenesis in the dentate gyrus, and the HPA axis [46], [47]. These changes can be viewed as epigenetic factors that alter the ability of the organism to cope with stressful situations in adulthood, and affect vulnerability to diseases/pathological conditions [48]. We found a substantial gender effect in the outcome of PRS on anxiety-like behavior assessed in the EPM, with females showing reduced anxiety and males, increased anxiety. While the increase in anxiety in PRS males is consistent with previous findings [1], [9], the reduction in anxiety that we observed in PRS females is in disagreement with the anxiogenic effect reported by Bowman et al. [7] using the open field test, and by Zagron and Weinstock [6] using the EPM. The different strains of rats (Wistar vs. Sprague-Dawley) and the different maternal stress paradigm used (one daily stress session of 30 min vs. three daily stress sessions of 45 min) may account for the differences in the EPM data obtained by Zagron and Weinstock [6] and our own. The possibility that the frequency and duration of prenatal stress affects not only the extent but also the direction of the behavioral outcome may have interesting preclinical and clinical implications, and is worth investigating. The contrary effects of PRS on anxiety in male and female rats, at least under our stress paradigm, are supported by the opposite changes in mGlu1/5 receptor function found in the ventral hippocampus, a region that is critically involved in risk assessment behavior in the EPM [49]–[56; but see also 57]. mGlu1 and mGlu5 receptors are established drug targets for the experimental treatment of anxiety [20], [58]–[64]. However, it is the blockade of these two receptor subtypes that relieves anxiety, and, therefore, the changes in receptor function that we observed are not in line with behavioral changes in male and female PRS rats. It is possible that the decrease and increase in mGlu1/5 receptor function observed in the ventral hippocampus of male and female rats, respectively, after PRS, represent an unsuccessful homeostatic mechanism aimed at restoring the “physiological” levels of the anxiety response. Alternatively, the reduced PI response to group-I mGlu receptor agonists in the hippocampus of PRS male rats might reflect a selective hypofunction of a restricted population of mGlu5 receptors that are present in neuroprogenitor cells and that support cell proliferation and survival [30]. If so, there might be a link between the reduced activity of mGlu5 receptors and the lower number of BrdU^+^ cells, with the ensuing reduction in the generation of new nerve cells [see also 5], [12] in the ventral hippocampus of male PRS rats. This may account for the increased anxiety of male PRS rats, if one gives credit to the hypothesis that anxiety is associated with reduced neurogenesis in the dentate gyrus [65]–[70]. Studies on mGlu1 and mGlu5 receptor knockout mice may help to clarify the specific contribution of these receptor subtypes to the behavioral outcome of PRS in males and females; however, this awaits the development of the PRS model in mice.

We found a reduction in mGlu2/mGlu3 receptor protein(s) in the hippocampus of both male and female PRS rats. This was the only parameter in which the consequences of PRS were uniform in the two sexes. mGlu2 and mGlu3 receptors are coupled to Gi proteins and produce pleiotropic intracellular effects, including the inhibition of cAMP formation, activation of the mitogen-activated protein kinase and the phosphatidylinositol-3-kinase pathways, and the amplification of mGlu1/5 receptor-mediated PI hydrolysis [17]. We have focused exclusively on the latter effect in the hippocampus by the use of either the selective mGlu2/3 receptor agonist LY379268 (combined with DHPG, a selective group-I mGlu receptor agonist) or the preferential mGlu2/3 receptor antagonist LY341495 (combined with 1S,3R-ACPD, a non subtype-selective mGlu receptor agonist). In male PRS rats, this component was greater than in control male rats, and overcame the reduction in mGlu1/5 receptor-mediated PI hydrolysis; in contrast, the mGlu2/3 component was stable or even lower in PRS female rats (compare responses to 1S,3R-ACPD and 1S,3R-ACPD+LY341495 or to DHPG and DHPG+LY379268 in Fig. 5). The activation of mGlu2/3 receptors relieves anxiety in rodents, and mGlu2/3 receptor agonists are being developed clinically for the treatment of generalized anxiety disorders and panic attacks [21]. The gender effect of PRS on anxiety is difficult to reconcile with changes in the expression and function of mGlu2/3 receptors in the hippocampus; however, it is hard to search for a correlation without dissecting the individual roles of these two receptor subtypes and examining all pathways activated by the two receptors.

The overall effect of PRS on spatial learning was less striking than the effect on anxiety, and was influenced by a sex difference in the performance of control rats in the water maze. However, PRS selectively influenced learning in female rats, improving their performance and eliminating the difference with male rats. Others have found a reduction in the performance of male PRS rats in the water maze using different paradigms of maternal stress [5], [6]. The improving effect of PRS on spatial learning in female rats was examined in the context of changes in hippocampal neuroplasticity involving changes in BDNF levels, neurogenesis, and group-I mGlu receptors. BDNF levels are directly correlated with the animal's performance in the water maze [71]–[78], and spatial learning induces BDNF production/expression in the hippocampus [79]–[81]. Adult hippocampal neurogenesis has been linked to the formation and consolidation of spatial memory [5], [82]–[96], and BDNF supports neurogenesis and is required for the long-term survival of newborn hippocampal neurons [32]–[35], [97], [98]. Curiously, adult male rats subject to PRS displayed no changes in spatial learning, confirming previous data where memory deficits were observed only during aging [8]. Male PRS rats also displayed a reduction in the survival of neuroprogenitors in the dentate gyrus, and an increase in hippocampal BDNF and pro-BDNF levels, the latter being indicative of enhanced BDNF expression/transcription/production. In contrast, female PRS rats showed no changes in neurogenesis or BDNF levels in spite of their improved performance in the water maze. A possible explanation for these data is that the positive feed-back loop involving hippocampal BDNF levels, neurogenesis, and spatial learning is disrupted in PRS rats. For example, the increase in BDNF levels in male PRS rats might have been developed as an adaptation aimed at avoiding learning impairments in spite of the reduction in neurogenesis. The only parameter that apparently correlates with improved learning in PRS female rats is the amplification of DHPG-stimulated PI hydrolysis in the dorsal hippocampus, which reflects an increase in the activity of group-I mGlu receptors. The activation of mGlu5 receptors in particular contributes to the induction of long-term potentiation (LTP) of excitatory synaptic transmission, a putative electrophysiological substrate for associative learning, in the hippocampus [99]–[105]. Mice lacking mGlu5 receptors exhibit defective LTP at the Schaffer collateral-CA1 pyramidal cell synapses, and are poor learners in the water maze [106], [107]. Thus, female PRS rats might learn more rapidly in the maze because of the increased efficiency of mGlu5 receptors in stimulating PI hydrolysis and inducing long-lasting changes in synaptic activity in the hippocampus.

In conclusion, we have shown a pronounced gender effect in the outcome of PRS on spatial learning, anxiety, and a number of neurobiological parameters that are classically correlated to hippocampus-dependent behaviors. However, this correlation is lost in PRS animals, where the direction of changes in the neurobiological parameters is unexpected and often counterintuitive. It has been shown that, in general, females are more resistant than males to stress-induced impairment of spatial tasks [116], a finding supported by our present results. Epigenetic modifications induced by PRS might perturb the physiological interplay among glutamate receptors, neurotrophic factors, and neurogenesis in the hippocampus, resulting in a series of adaptive mechanisms that are not apparently consistent with behavioral changes. Why this disruption results in the improvement of learning in female PRS rats is an interesting question that remains to be answered.

## Materials and Methods

### Animals

Nulliparous female Sprague-Dawley rats weighing approximately 250g each were purchased from a commercial breeder (Harlan, Italy). Animals were kept at constant temperature (22±2°C), with a regular 12hr light/dark cycle (lights-on at 8.00 a.m.). Water and food were available *ad libitum*. For a week after arrival, females were group-housed (4 per cage) to coordinate their estrous cycle. Females were then placed with a sexually experienced male for a night (the following day being designated as day 0 of gestation), after which they were housed individually in Plexiglas cages (30×20×15 cm). Pregnant females were then randomly assigned to perinatal restraint stressed (PRS) or control groups. (n = 12 in each group).

### Stress procedure

PRS was carried out according to our standard protocol [108]: from day 11 of pregnancy until delivery, pregnant females were subjected to three daily stress sessions starting at 09:00, 12:00 and 17:00 h, during which they were placed in transparent plastic cylinders (diameter = 7 cm; length = 19 cm) and exposed to bright light for 45 min. Control pregnant females were left undisturbed in their home cages. Male and female offspring were weaned 21 days after birth, and only male offspring from litters containing 10–14 pups with a comparable number of males and females were used for the experiments. A maximum of two male or female pups were taken from each litter. After weaning, male and female rats from each experimental group (control and PRS) were housed in groups of three and maintained under similar environmental conditions until the experiments were started (3 months of age). All experiments followed the rules of the European Communities Council Directive 86/609/EEC.

### Behavioral tests

Eight to ten animals per group (control and PRS, males and females) were used for behavioral analysis.

#### Elevated Plus Maze

The Elevated Plus Maze (EPM) consisted of two open illuminated arms (50×10 cm) and two enclosed arms of the same size with 40-cm-high walls, placed so that identical arms (either open or closed) lie opposite each other, with a 10 cm^2^ platform at the intersection of the arms. The device was made of lacquered wood and placed at a height of 50 cm from the floor, a standard height [109], [110] aimed at limiting the attempts of the animals to escape from the apparatus. The animal was placed on the platform in the middle of the maze facing an open arm, and the number of entries and time spent (with all four paws) inside each arm were manually scored for 5 minutes. Total locomotor activity was assessed by monitoring the total number of entries into an arm (opened or closed) [see 110]. The device was cleaned with 10% ethanol after each trial to effectively remove the scent of the previously tested animal.

#### Morris Water Maze

The apparatus consisted of a circular pool (diameter 200 cm, height 60 cm) located in a test room with white walls with several cues on them. The pool, with its inner surface painted black, was filled to a depth of 40 cm with water (maintained at 25±1°C), covering an invisible (black) 10-cm square platform. The platform was located approximately 2 cm below the surface of the water. The pool was virtually divided into 4 quadrants and the platform placed at a fixed position in the centre of a quadrant. Rats underwent 5 trial training sessions separated by 24 h for five consecutive days. For each trial, rats were allowed to swim until they reached the escape platform and climbed onto it. A cut-off of 90 sec was chosen, at the end of which the animal was placed on the platform and left there for a reinforcement period of 30 sec. The starting position of the animal varied, avoiding the quadrant of the platform in each trial. A video camera above the centre of the pool was connected to a computerized tracking system that recorded and analyzed animal behavior (San Diego Instruments). Escape time and swim speed were measured.

### Assessment of neurogenesis

Three month-old animals were injected 2 times a day (at 11:00 and 15:00 hrs) for five days with the thymidine analog bromodeoxyuridine (BrdU, 75 mg/kg i.p.) to label dividing cells. Animals were killed two weeks after the last BrdU injection.

#### Immunohistochemistry

Rats (five per group) were anesthetized with sodium pentobarbital (60 mg/kg) and perfused with 200 ml saline (NaCl, 0.9%, w/v), followed by 400 ml of cold phosphate buffer (PB, 0.1 M, pH 7.4) containing 4% paraformaldehyde. Samples from all groups were processed in parallel to avoid any non-specific effect of the staining procedure. Twenty-four hours after postfixation in paraformaldeyde, brains were transferred to 0.1 M PB solution and stored until processed for immunohistochemistry or immunofluorescence. Serial brain sections(40 µm thick) were cut through the hippocampus using a vibratome (Leica, France) and collected in 0.1 M PB. Free-floating sections were processed using a standard immunohistochemical procedure. DNA denaturation was carried out by incubation for 20 min in 2N HCl at 37°C followed by two rinses in 0.1 M borate buffer (pH 8.5). Following several rinses in 0.1 M PB, the sections were incubated for 45 min in 3% normal donkey serum (NDS)/ 0.1% Triton X-100 in 0.1 M PB and then incubated for 48h at 4°C with anti-mouse BrdU (1∶500, Boehringer Mannheim, Indianapolis IN) diluted in 1% NDS/0.1 M PB. Sections were then incubated for 2h with the secondary antibody (1∶500, biotinylated donkey anti-mouse) diluted in PBS-Tds. After washing in 0.1 M PB, sections were incubated for 1hr with avidin-biotin-peroxidase complex (ABC Elite kit, Vector laboratories) and rinsed in 0.1 M PB. Peroxidase was detected using the glucose oxidase-DAB-nickel method [111]. Sections were rinsed with water, then washed repeatedly with 0.1 M PB, mounted onto gelatin coated slides, air-dried for 30 min, dehydrated in ethanol, cleared in xylene and coverslipped using Eukitt (Germany).

Using markers for mature neurons (NeuN) and astrocytes (GFAP), we determined the phenotype of BrdU-immunoreactive (BrdU^+^) cells. For BrdU^+^/NeuN^+^ and BrdU^+^/GFAP^+^ labeling, the sections were pre-treated as above, then incubated with a cocktail of rat anti-BrdU (1∶500, AbCys, France) and mouse anti-NeuN (1∶1.000, Chemicon, France) or rabbit anti-GFAP (1∶500, Dako, France) for 24 h at 4°C. After rinsing, the sections were exposed for 2h to the appropriate fluorescent secondary antibodies (Alexa 488-conjugated goat anti-mouse, Molecular Probe, TRITC-conjugated donkey anti-rat, Jackson, and FITC-conjugated goat anti-rabbit, Sigma, France) at room temperature. The sections were coverslipped in Mowiol mounting medium and then visualised using a confocal microscope (Zeiss, LSM 510, France).

#### Quantification

All analysis was performed in a blind manner using coded sections. Every sixth section in a series of 40 µm thick sections was analyzed: an average of 18 sections was analyzed from each animal. All BrdU^+^ nuclei in the subgranular zone (SGZ) and in the granule cell layer (GCL) of the dentate gyrus (DG) were counted bilaterally under the microscope using 40× and 80× objectives. Quantification was carried out over the two hemi-hippocampi and expressed as the sum of the BrdU^+^ cells in the SGZ and GCL.

The number of BrdU^+^ cells was examined separately in the rostral (−1.8 to −3.8 from bregma) [112] and caudal hippocampus (−4.16 to −6.3 from bregma). The rostral region contains the dentate gyrus in most of the dorsal hippocampus, whereas the caudal region contains a part of the dentate gyrus in the dorsal hippocampus and the entire dentate gyrus in the ventral hippocampus. The total number of BrdU^+^ cells in the dorsal and ventral regions was obtained from the number of BrdU^+^ cells observed in 9 sections per rat.

The percentage of double-labeled cells (BrdU^+^/NeuN^+^, BrdU^+^/GFAP^+^) was determined by confocal laser microscopic analysis using a 60× oil objective. For each rat, 4 sections located in representative regions for the dorsal and ventral hippocampus were analyzed throughout the z-axis with a 1 µm step in order to exclude false double-labeling, for a minimum of 60 BrdU^+^ cells.

### Measurement of Polyphosphoinositide hydrolysis

Stimulation of polyphosphoinositide (PI) hydrolysis by mGlu receptor agonists was examined by measuring the accumulation of [^3^H]-inositolmonophosphate ([^3^H]InsP) in hippocampal slices, as described previously by Nicoletti et al. [113]. Five animals per group (control and PRS, males and females) were used and analyses performed in duplicate. Animals were killed by decapitation and the hippocampi dissected. To differentiate between the dorsal and ventral regions, the entire hippocampus was placed flat on a cooled surface and divided into equal dorsal and ventral parts [114]. The whole hippocampus or separate dorsal and ventral hippocampi were dissected and cut into 350×350 µm slices with a McIlwain tissue chopper (Mickle laboratory Engineering Co., Inc., Gromshell, UK). Slices were incubated at 37°C under constant oxygenation for 30–45 min in Krebs–Hensleit buffer equilibrated with 95% O_2_, 5% CO2 to pH 7.4. An aliquot of the slices was then incubated with 0.3 mM myo[2–^3^H]-inositol (Amersham, specific activity 10 Ci/mmol) to label inositol phospholipids. At the end of this incubation LiCl (10 mM) was added, followed 10 min later by different combinations of a non-subgroup-selective mGlu receptor agonist, ACPD (1S,3R-aminocyclopentane-1,3-dicarboxylic acid) at 100 µM, a selective group-I mGlu receptor agonist, DHPG, at 200 µM, a selective group-II agonist, LY379268 at 1 µM, and a selective group-II antagonist, LY341495 at 1 µM. Sixty minutes later, slices were washed three times with an excess of ice-cold buffer containing 0.4 mg/ml LiCl, and the reaction was stopped with 900 ml of methanol/chloroform (2∶1). After the further addition of 300 ml chloroform and 600 ml water, the samples were centrifuged at low speed to facilitate phase separation, and the upper aqueous phase was loaded onto columns containing Dowex 1-X-8 resin (format form, 100–200 mesh, Bio-Rad). [^3^H]InsP was eluted with 6.5 ml of a solution containing 0.2 M ammonium formate and 0.1 M formic acid, and quantified as described previously [113]. Protein content was measured as described by Lowry et al. [115].

### Western blot analysis

Seven animals per group (controls and PRS, males and females) were used and analyses performed in duplicate. Rats were killed by decapitation and brains rapidly removed; hippocampi were dissected and stored at −80°C. On the day of the experiment, tissue was homogenized at 4°C with a polytron in 500 µl of 100 mM Tris buffer, containing phenylmethylsulfonyl fluoride 1 mM, leupeptin 10 µg/ml and aprotinin 10 µg/mL pH 7.2. Protein concentrations were determined using the Bradford protein assay. Fourty micrograms of protein were resuspended in sodium dodecyl sulfate (SDS)-bromophenol blue reducing buffer with 40 mM dithiothreitol. The samples were separated on 8% (for mGluR analysis) or 12% (for BDNF analysis) SDS-polyacrylamide gels (Amersham Bioscience, Inc., Little Chalfont, England) and after electrophoresis (Mini-PROTEAN 3 System, Bio-Rad, Hercules, CA, USA), the proteins were transferred to nitrocellulose membranes (Amersham Bioscience) using 35 mM Tris, 192 mM glycine and 20% methanol for 4 h. Samples from male and female rats underwent electrophoresis separately but for each gender, samples from control and PRS rats migrated on the same gel. After transfer, blots were incubated in a solution (blocking solution) containing Tris-buffered saline (TBS), 0.5% (w/v) Tween-20, 1% (w/v) non-fat milk and 1% (w/v) bovine serum albumin. Subsequently, blots were incubated overnight with rabbit anti-mGluR1 (1∶500), rabbit anti-mGluR5 (1∶1000), mGluR2/3 (1∶1000) (Upstate Biotechnology, Lake Placid, NY, USA), or rabbit anti-BDNF (1∶500, Santa Cruz, USA) in blocking solution at 4°C. After incubation with the primary antibody, the blots were incubated with horseradish peroxidase-conjugated goat anti-rabbit antibodies (1∶10.000; Amersham Bioscience) for 1h at room temperature (21°C±2). To ensure that each lane was loaded with an equivalent amount of protein, the blots were probed with an anti-actin serum (1:1000; Sigma, St Louis, MO, USA) overnight at 4°C. Subsequently, blots were incubated with horseradish peroxidase-conjugated goat anti-mouse antibodies (1∶5000; Amersham Bioscience) for 1h at room temperature. Immunoreactive bands were visualized with an enhanced chemiluminescence system (Amersham Biosciences). After immunoblotting, digitized images of bands immunoreactive for target (mGluR1 or mGluR5 or BDNF) and control (actin) molecules were acquired and the area of immunoreactivity corresponding to each band was measured using the Scion Image computer program (Scion Corp., Frederick, MD, USA). A ratio of target to actin was then determined, and these values were compared for statistical significance.

### Statistical analysis

Data on EPM, neurogenesis, and PI hydrolysis in the hippocampus as a whole were analyzed using two-way analysis of variance [ANOVA; 2 groups (control and PRS)×2 sexes]. Results of PI hydrolysis by the dorsal and ventral part of the hippocampus were analyzed with two-way ANOVA (2 groups×2 regions) separately for males and females, since it was not feasible to have more than two variables in the same experiment.. Immunoblot data were analyzed separately for males and females with one-way ANOVA. Latencies in the Morris water maze were analyzed by four-way ANOVA for repeated measures [2 groups×2 sexes×5 trials×5 days with repeated measures on trials and days]. The ANOVA analyses were always followed by Newman-Keul's *post-hoc* comparisons. The level of significance was set at p<0.05.
